# The Gracilis Muscle Flap: A “Work Horse” Free Flap in Diabetic Foot Reconstruction

**DOI:** 10.29252/wjps.10.2.33

**Published:** 2021-05

**Authors:** Skanda Shyamsundar, Ali Adil Mahmud, Vishal Khalasi

**Affiliations:** 1 Head of department plastic surgery, Kauvery hospital, Trichy, Tamil Nadu, India; 2 Consultant plastic surgeon, department plastic surgery, Kauvery hospital, Trichy, Tamil Nadu, India

**Keywords:** Gracilis free flap, Diabetic foot, Microvascular reconstruction

## Abstract

**BACKGROUND:**

Diabetes is a leading cause of foot ulcers and lower limb amputation throughout the world. Adequate wound debridement and cover is the standard of care, but lack of adequate vascularised local tissue poses a major challenge. The gracilis flap offers various advantages in this respect, which we would like to discuss in this study, and hence makes it an attractive option in diabetic foot patients.

**MATERIAL AND METHODS:**

This retrospective study was conducted over a period of 2 years, from 2018 to 2020 in the Department of Plastic Surgery, Kauvery Hospital, Trichy, India. The flap harvest time, total operation time, flap take and complications associated with the procedure were noted.

**RESULTS:**

Overall, 56 patients were enrolled. The average flap harvest time was 55 +/- 10 min and the average overall operation time was 240+/- 30 minutes. There was complete flap survival in 42 (75%) patients, a partial survival in 12 (21.42%) patients and complete flap loss in 2 (3.57%) patients. In the donor site complications hypertrophic scarring was reported in 5 (8.92%) and donor site seroma in 3(5.3%) patients.

**CONCLUSION:**

The free gracilis flap offers good wound healing and excellent foot contour besides being safe and effective in small to medium sized defects makes it an excellent free flap in diabetic foot reconstruction.

## INTRODUCTION

Diabetes is a leading cause of lower extremity ulcers and amputations worldwide. About 83% of all non-traumatic foot amputations are associated with diabetes^1^. After one limb affection, there is a 20-50% chance of amputation in the contralateral limb^2^^-^^5^. The 5-year mortality rate in these patients after one amputation can be as high as 70%^6^^,^^7^.

Adequate and aggressive debridement, glycaemic control and wound cover is the standard treatment for these patients. While many patients can get away with a simple skin graft ^8^, exposure of bone, tendon or nerves mandates a flap cover.

Various locoregional flaps have been described but the presence of chronic inflammation, fibrosis and lack of adequate vascularised local tissue is an important limiting factor in doing so.

Microvascular free tissue transfer to the wound site can induce angiogenesis and accelerate wound healing even in hypovascular wounds^9^. Various microsurgical free fascio-cutaneous and muscle flaps are described in literature^10^^-^^14^, but some of the major reasons why surgeons still avoid free flaps are the long operation time, condition of the recipient vessels and the added stress to a diabetic patient who usually has other associated comorbidities.

At our institute, we handle a large number of patients with diabetic foot ulcers and routinely use the gracilis muscle-free flap with an operating time almost similar to a pedicled flap with encouraging results. Although we have used most of the named free flaps for lower limb reconstruction, in this study we discuss why the gracilis flap is our “go-to” free flap in diabetic foot reconstruction.

## MATERIAL AND METHODS

This retrospective study was conducted in the Department of Plastic Surgery, Kauvery Hospital, Trichy, India, over a period of 2 years from 2018-2020. The inclusion criteria were: 1) all patients of type 1 and type 2 diabetes mellitus with small to medium defects of their foot requiring a flap cover; 2) patients with ulcers on weight-bearing areas of foot; 3) patients with a palpable pulse (Dorsalis Pedis or Posterior Tibial) as a pre-operative angiogram was not routinely conducted in our patients.

Exclusion criteria were patients not fit for surgery, not having a palpable recipient artery pulse or not giving consent for photography. All the surgeries were conducted by a single surgeon (S.S). Total operation time, flap harvest time, flap success, donor and recipient site complications and ability to wear footwear were recorded. 

All the foot ulcers were graded according to Wagner’s classification^15^, patients were initially admitted, blood sugar and nutrition was improved, pus culture was taken and antibiotics were started. Patients were taken up for early surgical debridement with or without NPWT and garcillis flap cover was done within the first 7-10 days of admission. A 2-surgeon approach was used to reduce operation time, a pure muscle flap was taken in all our cases, no skin paddle was included and the muscle was covered with a split skin graft. The recipient's vessels were either the Anterior Tibial Artery/ Dorsalis Pedis artery (end-to-end anastomosis) or the Posterior Tibial Artery (end to side anastomosis). 2Consultant Plastic surgeon, Department of Plastic Surgery Kauvery Hospital, Trichy, Tamil Nadu, India. 

This study was approved by our hospitals Ethical Committee, and informed consent was taken from all the patients.

Patients were divided into three groups, 1) Complete survival: complete flap and graft take after at least 6 months postoperatively. 2) Partial survival: partial flap or graft loss with a draining wound not healed within 6 months of surgery and 3) Complete failure: complete loss of the flap or graft within 6 months of procedure requiring a repeat procedure. 

## RESULTS

Overall, 56 patients were enrolled, of whom 54 (96.42%) were male and 2 (3.57%) were female. Forty (71.42%) patients were >50 yr age, 11 (19.64%) patients were between 25-50 yr and 5 (8.92%) patients were < 25 yr age, with the oldest patient being 63 yrs old and the youngest being 21 yrs old. Most of the patients, 51 (91.07%) suffered from type II diabetes while 5 (8.92%) suffered from type I diabetes mellitus.

**Fig. 1:**
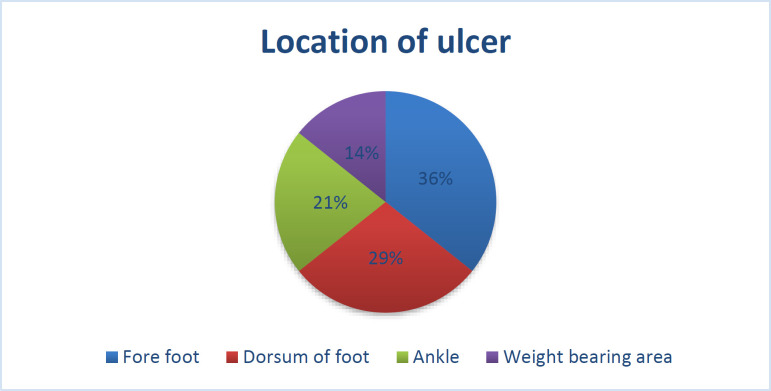
Location of the ulcer

The most common location of ulcer was on the forefoot in 20 (35.71%) patients, dorsum of foot in 16 (28.57%), the ankle joint region in 12 (21.42%) and the heel pad or weight-bearing area of the foot in 8 (14.28%) patients (Figure 1). Most of the patients, 22 (39.28%) had a Wegner’s type III ulcer while 18(32.14%) and 16 (28.57%) patients suffered from type IV and type II ulcers respectively.

We had complete flap survival in 42(75%) patients, a partial flap survival in 12(21.42%) patients and complete flap loss in 2 (3.57%) patients. In the donor site complications, hypertrophic scarring was reported in 5 (8.92%) patients and seroma in the donor site was in 3 (5.35%) patients. The follow-up period was 12 +/- 6 months.

***Case 1***

A 45-year-old man presented with an infected diabetic foot ulcer on the sole and weight-bearing area of the heel (Figure 3a). The patient was admitted and after adequate debridement and glycaemic control and a free gracilis muscle flap was performed with an end to side anastomosis to the posterior tibial artery with a skin graft used to cover the flap (Figure 3b, Figure 3c). The flap settled well with a good foot contour (Figure.3d, Figure. 3e). 

**Fig. 2:**
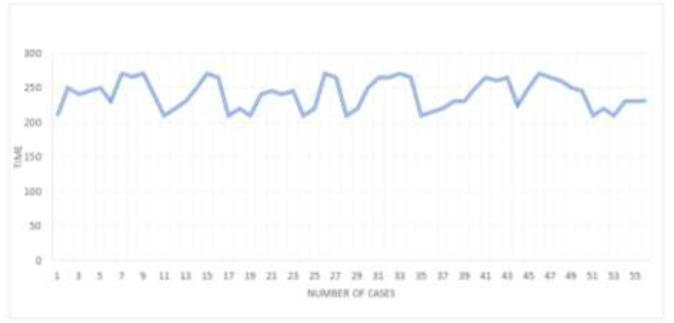
Total operation time (Min) Vs number of cases

The flap harvest time ranged from 55 +/- 10 min and the overall operation time was 240+/- 30 min [4 hours +/- 30 min] (Figure 2). The Anterior Tibial/ Dorsalis Pedis artery was used as the recipient vessel in 46(82.14%) patients while the posterior tibial artery was used in 10(17.85%) patients.

**Fig. 3a:**
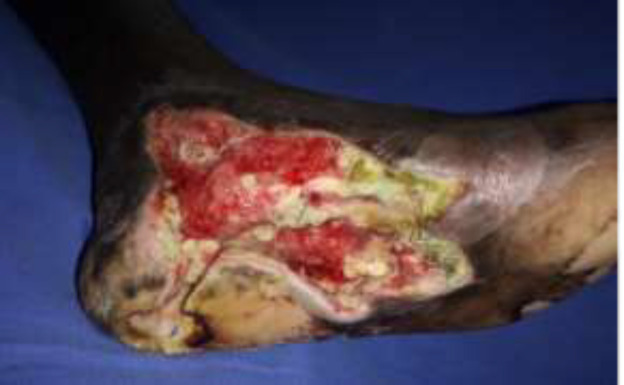
Left foot diabetic ulcer

**Fig. 3b:**
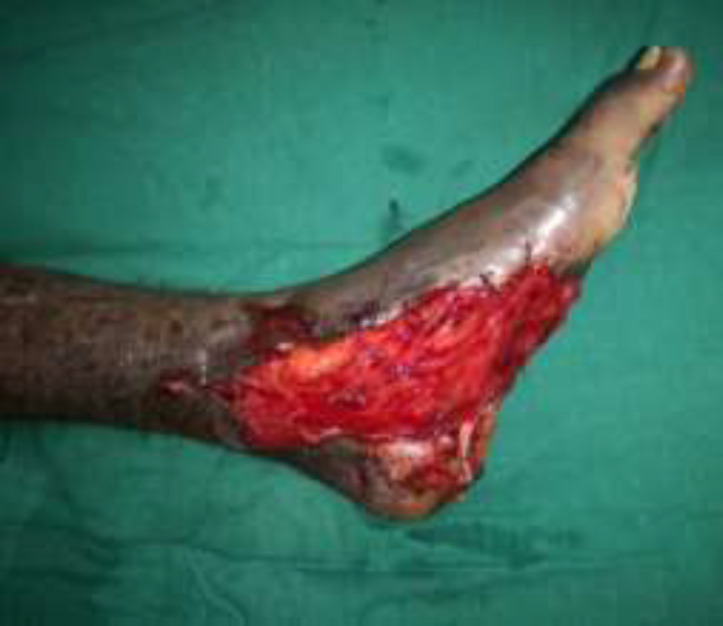
Free Gracilis muscle flap attached to ulcer

**Fig. 3c:**
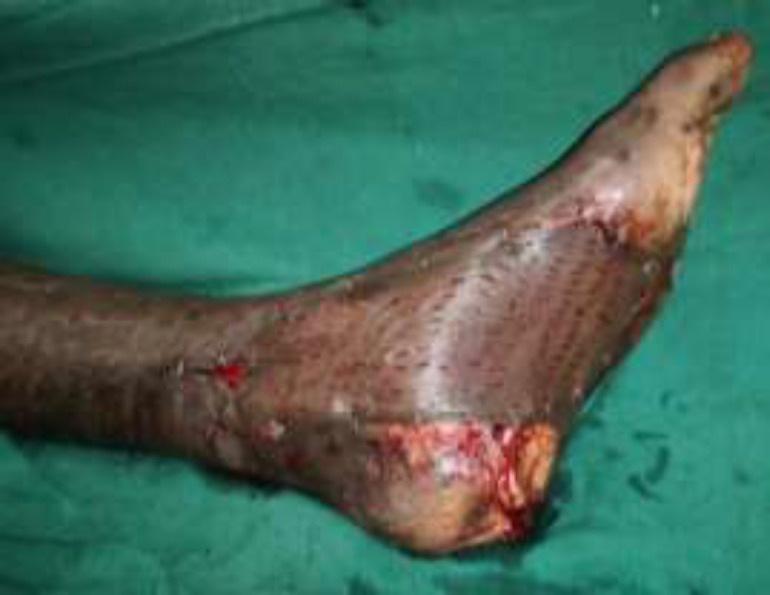
Flap covered with split-thickness skin graft

**Fig. 3d:**
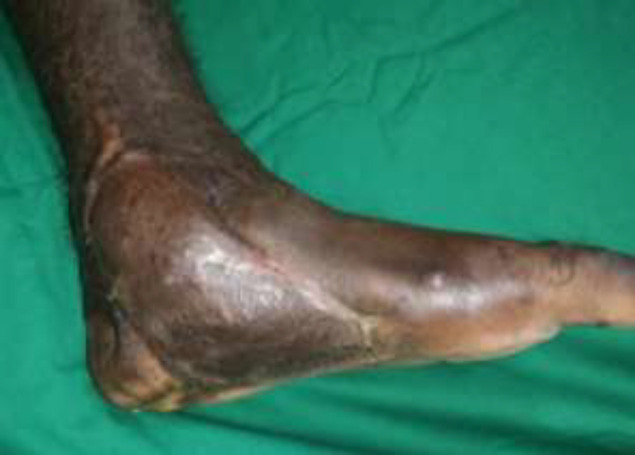
After 4 months follow up well-settled flap

**Fig. 3e:**
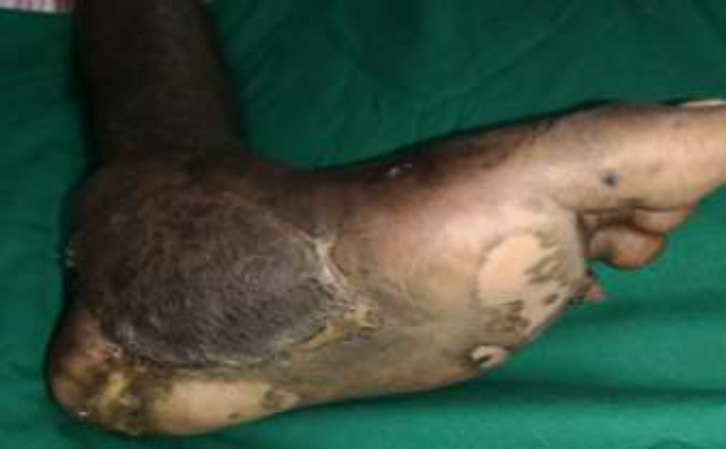
After 4 months follow up well-settled flap

***Case 2***

A 30-year male presented with a diabetic foot ulcer exposing the ankle joint (Figure 4a). After debridement, a Gracilis flap was placed with end-to-end anastomosis to the anterior tibial artery and a skin graft applied (Figure 4b and 4c). Post operatively at 4 months follow-up showd a well-settled flap with excellent foot contour and graft take (Figure 4d).

**Fig. 4a:**
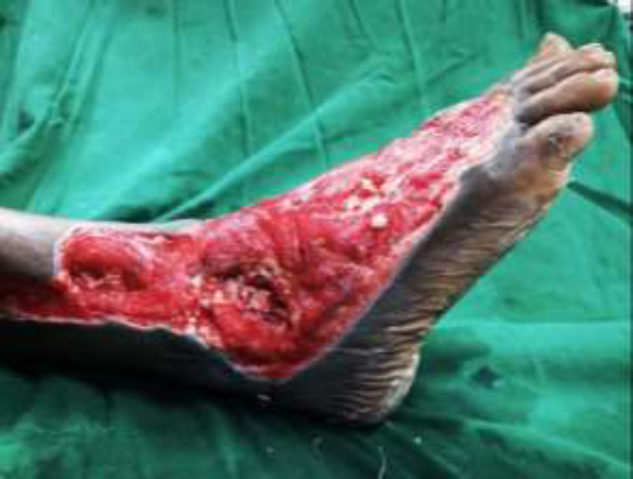
Right foot ulcer with exposed ankle joint

**Fig. 4b:**
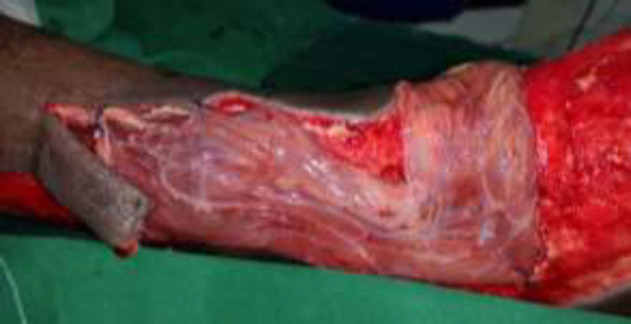
After gracilis flap cover

**Fig. 4c:**
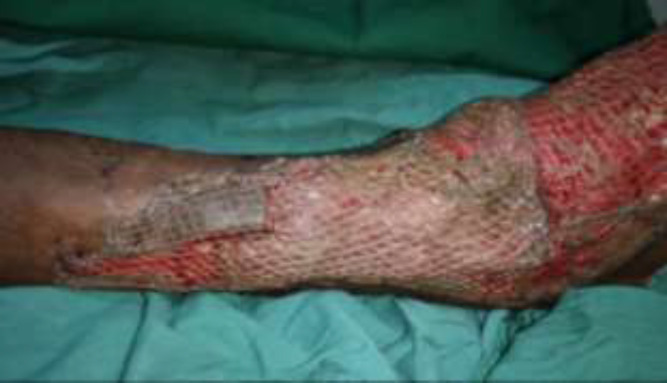
After split thickness skin graft

**Fig. 4d:**
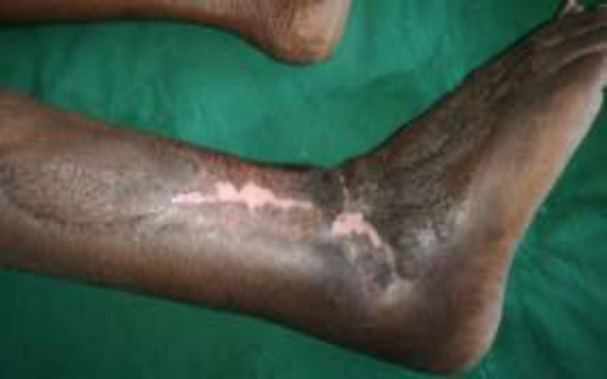
Four months post-operative, excellent foot contour

***Case 3***

A 55-year-old man presented with a badly infected diabetic foot ulcer to the right foot (Figure 5a). After debridement and gracilis flap cover (Figure 5b and 5c) with end to side anastomosis to the posterior tibial artery, the patient had a good graft take and excellent foot contour (Figure 5d).

**Fig. 5a:**
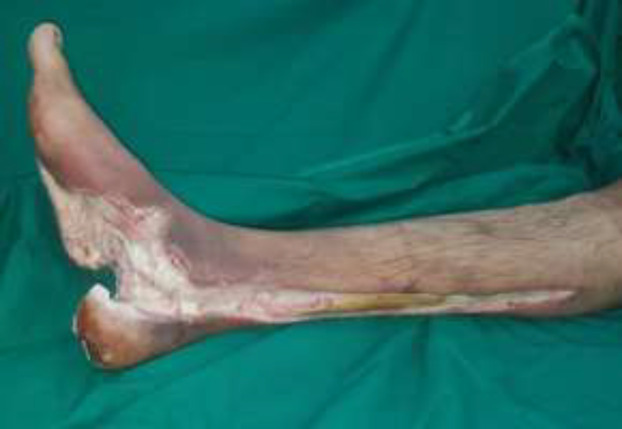
Right foot infected ulcer

**Fig. 5b:**
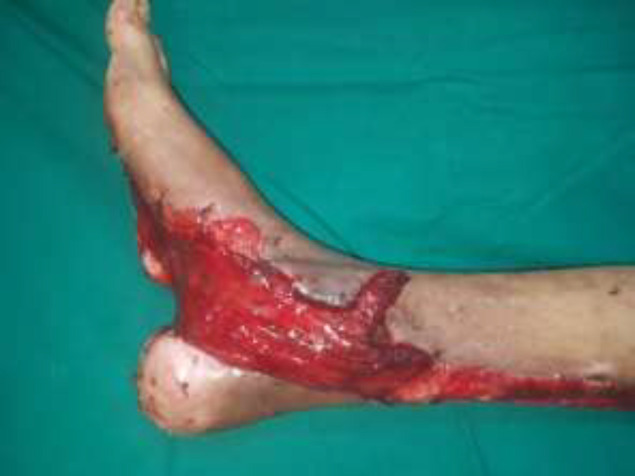
After gracilis flap cover

**Fig. 5c:**
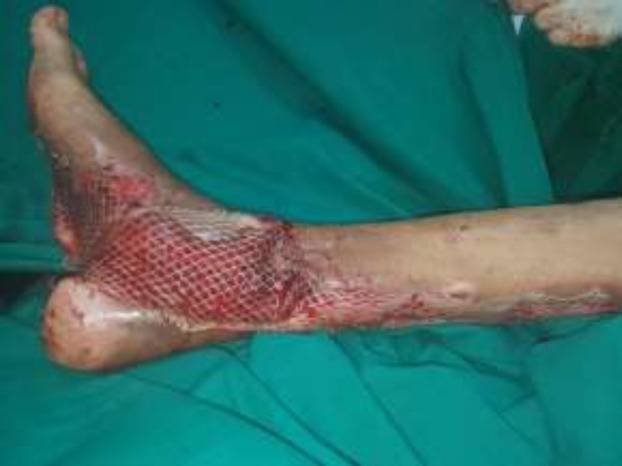
Flap covered with skin graft

**Fig. 5d:**
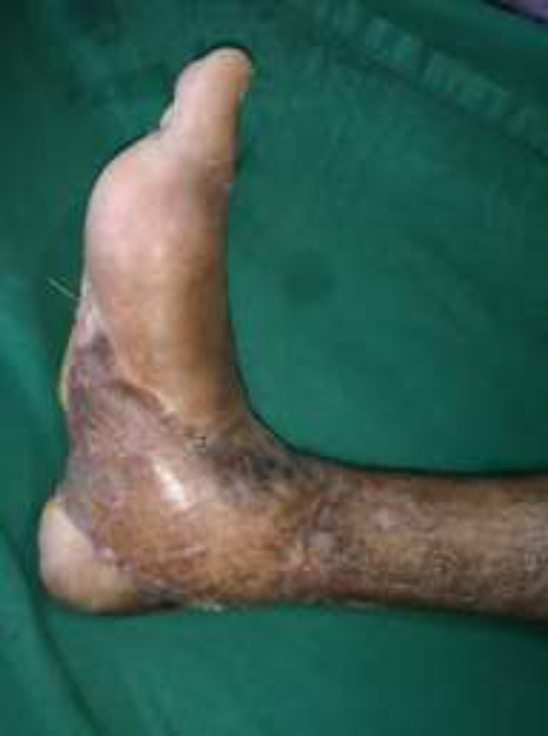
6 months post operative, excellent foot contour

## Discussion

Diabetes is one of the leading causes of lower limb ulcers leading to avoidable amputations, with the numbers on a steady rise this is a serious cause for concern. Chronic high blood sugar alters the intracellular myoinositol sorbitol pathways, which predisposes to neuropathy^15^ this, in turn, leads to a loss of the body’s protective sensation to trauma and pressure. The combination of diminished sensation and blood supply along with raised blood sugars and decreased immunity makes these patients highly susceptible to infections and wounds. Aggressive treatment is warranted in all these patients with antibiotics, cultures, surgical debridement and decompression along with effective and stable wound cover. 

With the onset of microsurgery, the ability to transfer vascularised tissues to the wound site has greatly improved wound healing and improved wound salvage rates^16^^-^^18^. In a study of 45 diabetic patients, free flaps improved wound healing and neovascularization was reported in these ischemic ulcers^19^.

The gracilis flap offers several favourable advantages for use such as:

1) Quick and easy dissection with a lower learning curve allowing a two-surgeon approach.

2) Minimal donor site morbidity and no loss of function, 

3) Muscle flaps have the added advantage of filling in the dead spaces of the wounds and bone with vascularised tissue.

4) The major advantage in our view is that once the muscle atrophies it takes the shape and contour of the foot facilitating footwear and no secondary debulking is required.

The major disadvantage of the gracilis is:

1. The short pedicle length and vessel diameter, mandating the use of the microscope for anastomosis, as compared to the ALT which many surgeons are even comfortable doing under loupe magnification.
2. The smaller muscle size as compared to the Latissimus dorsi flap necessities its use only in small to medium-sized tissue defects.

Some muscle flaps like the Latissimus dorsi have certain unfavourable characteristics such as: changing the patient’s position intraoperatively, while in the rectus abdominis muscle flap the chance of herniation and mesh infections are present. The use of the rectus femoris muscle flap was demonstrated for free tissue transfer with various advantages but one of the major disadvantage is the lack of the ability of the muscle to spread and increase in surface area as compared to a gracilis flap, we found that a gracilis flap can be easily spread to twice or thrice its width^20^.

Fasciocutaneous flaps like the antero lateral thigh flaps take a relatively longer time for dissection as compared to the gracilis and may also requires secondary debulking to facilitate footwear use. Omer Ozken et al^21^ discussed the reliability of free flaps in diabetic foot reconstruction in 13 patients, they used fasciocutaneous and muscle flaps but favoured the former because of better tissue match and the ease of post-operative monitoring. In all our patients, we were very comfortable with the gracilis flap, post-op monitoring was not an issue as we noted the muscle colour and bleed through the skin graft fenestrations. None of our patients needed secondary debulking as the flap atrophied well and took the contour of the foot.

The flap harvest in our study was 55 +/- 10 min, this greatly decreased the overall operating time (4 hours +/-30 min) and hence was a great advantage in diabetic patients with associated comorbidities like heart disease who were not good candidates for prolonged procedures. The primary closure of the donor site was also speedy as there was no tissue loss and the patients were left with only a single linear scar.

A study^22^ on 45 patients with diabetic foot ulcers treated solely with a gracilis free flap reported a complete flap success rate of 72.9% which is similar to our 75% and had complete flap loss in 1 (2.1%) patient while we report a complete flap loss in 2 (3.57%) patients. All their patients reported a minimal donor site morbidity with only 3 (6.6%) patients complained of hypertrophic scarring, in our patients' donor site hypertrophic scarring was reported in 5 (8.92%) patients which settled with conservative treatment and seroma in the donor site was in 3 (5.35%) patients. 

## CONCLUSION

The free gracilis flap is a safe and effective free flap in small to medium-sized diabetic foot defects with minimal donor site morbidity and speedy flap harvest. The good wound healing and excellent foot contour offered by it without the need for secondary debulking procedures makes it our “work horse” free flap in diabetic foot reconstruction.

## CONFLICT OF INTEREST

None
